# Beyond identity and generations: bringing life course theory to studies of older gay men

**DOI:** 10.3389/fsoc.2024.1393607

**Published:** 2024-05-15

**Authors:** Dana Rosenfeld, Jesus Ramirez-Valles

**Affiliations:** ^1^College of Liberal Arts and Sciences, University of Westminster, London, United Kingdom; ^2^School of Medicine, Univeristy of California San Francisco, San Francisco, CA, United States

**Keywords:** life course theory, gay male aging, queer aging, life course, male aging

## Abstract

The last century’s numerous, rapid social changes affecting gay men make studies of gay male aging a ripe topic for life course theory, which views later life as the product of historical grounded interchanges between individual lives, social change, and structural contexts. That identifying as gay can occur at any point in the life course widens some life course theorists’ primary focus on early-life events to include those occurring throughout the life course. Yet most historically-attentive research on older gay men focuses on generations and identity development rather than on cohorts – groups who entered a system or context at the same time - or on the cumulative, concrete outcomes of encountering social change at a particular point in the life course. This article argues for gay male aging studies’ use of life course theory, specifically, its focus on cohort membership’s implications for later life, including cumulative disadvantage, in addition to more generationally-focused investigations. After briefly reviewing scholarship on older gay men, we introduce the life course approach and its critique by queer gerontologists for adopting a heteronormative view of the LGBT life course and eliding its distinctive contours. With particular attention to later-life concrete outcomes rather than identity formation, we explore key historical events in gay men’s lives that have produced (in the case of the AIDS epidemic) or could produce (for example, the Marriage Equality Act, the Don’t Ask, Don’t Tell policy) distinctive gay male cohorts. We then consider intra-cohort variation within gay male cohorts before exploring some the barriers to investigating cohorts and cohort effects among older gay men.

## 1 Introduction

The aging of the Baby Boomer generation has inspired growing academic and community-level interest in gay aging, with gay aging research becoming more theoretically informed, less marginalized, and more attuned to the complexities of aging as a member of one or more sexual minoritized group. Yet, while gay aging studies have been rightly attentive to generational shifts and differences, they have not yet taken full advantage of life course theory (but see Rosenfeld, 1999, 2003; Tester, 2018; Lopez-McCoy, 2021), a diverse area of study focused on ‘the historic interplay among people’s lives, structural contexts, and social change’ (O’Rand, 1996, p. 230) and less focused on generations than on cohorts – broadly defined, groups who entered a system or context at the same time. As Hareven (1994, p. 438) wrote, ‘Rather than viewing older people as a homogeneous group, a life course perspective considers them as age cohorts moving through historical time, each cohort with its distinct life experiences shaped by the circumstances encountered earlier in life’.

Life course theory frames aging and later life as the products of interplay. This interplay is consequential at the time these lives, contexts, and changes first intersect, but can also have long-term, cumulative effects (e.g., health, finances, quality of life, education) by interrupting or derailing typical life ‘trajectories’ or ‘pathways’ – ‘the course of an individual’s experiences in specific life domains (or spheres) over time’ – (Settersten, 1999, p. 19) – directed along the lines of a normative, institutionally supported life course. Disadvantaged groups are, by definition, more vulnerable to these interplays’ negative consequences and, as more advantaged groups are typically protected from them, these impacts drive cumulative disadvantage (increasing inequality between groups over time – O’Rand, 1996, p. 232). Thus, in life course theory, social change is much more central than it is in less historically-sensitive gerontological work which dominates the field.

Our purpose here is to further gay male aging research by considering how life course theory can uncover the mechanisms that lead to gay male aging’s distinctive qualities. Put simply, life course theory provides the tools needed to unpack factors shaping the lives of older gay men anchored in historical time, while cumulative disadvantage can link their concrete, later-life circumstances to earlier life events. We argue that while the life course approach’s focus on the long-term effects of social change is ‘always already’ of direct utility to aging studies overall, it is especially apposite for research into gay male aging, given the last 100 years’ rapid social changes in, *inter alia*, sexual and gender politics and in gay people’s legal protections or lack thereof (e.g., Hammack and Cohler, 2011).

We focus on gay male, rather than on lesbian, gay, bisexual, transgender, and queer (LGBTQ) aging for three reasons. The first is that, while we recognize that many of the instances of social change that we explore here affected both gay men and lesbians, the lesbian life course, and older lesbians’ history with feminism and lesbian-feminism (see, e.g., Traies, 2016), introduce such distinctive experiences that to consider them alongside older gay men would risk eliding factors that shape either group’s later lives. The second is the dearth of robust research into bisexual and transgender aging, although this area of research is growing (see, e.g., Fabbre, 2014; Witten, 2016; Fredriksen-Goldsen et al., 2017; Jen and Jones, 2019; Sloan and Benson, 2022). The third is the problematic nature of uncritically applying the term ‘LGBTQ’ – designed to signify the gay community’s new inclusiveness of a wider range of sexual and gender identities and practices – when researching aging among gay men who do not share many of the bisexual and transgender communities’ distinctive circumstances, challenges, and experiences (see also Fredriksen-Goldsen et al., 2017). As Westwood et al. (2020) argues, ‘Collectivized approaches to heterogenous identity-based groups address commonalities but often fail to address internal diversity’ (p. 1), and ‘Homogenizing language and terms bely the heterogeneity of individuals and sub-groups falling under the ‘older LGBT+’ umbrellas’ (p. 6). Similarly, given the relative preponderance of gay aging studies that focus on Western populations, we will focus on Western gay male aging research, primarily that conducted in the United States (US).

## 2 Core concepts and approach

Social gerontologists broadly agree about the causal significance of social factors for the aging experience but remain internally divided over which social factors to consider and how to do so. Gerontologists vary in the weight they give to the long-term effects of earlier life stages and experiences on later life itself: is aging the product of a life already lived, or is it a broadly universal experience regardless of historical contexts and events encountered in previous years?

While recognizing the roles of other factors in the aging process, life course theory explicitly adopts the first of those options. It views later life as shaped by such social statuses as sex, gender, ethnicity, racially minoritized status, and socio-economic class, by individuals’ agency, and by the normative, institutionalized life course that encourages and rewards adopting specific roles (e.g., spouse, parent, worker, retiree) at broadly similar ages/life stages, but as equally shaped by the immediate and long-term effects of encountering significant historical contexts and events at particular points in the life course. While many life course theorists have given early-life events more weight than they give events that occur later in the life course, others have critiqued this stance for eliding older people’s capacity for change in response to new events (see Dannefer and Settersten, 2010, p. 8).

Because the institutionalized life course structures individuals’ movements through broadly agreed-upon life ‘stages’, sharing a life stage (e.g., childhood, young adulthood, middle adulthood, older age) with one’s peers typically means sharing similar socially-supported and expected age-graded roles (e.g., student, new homeowner/parent, worker, retiree), circumstances, resources, constraints, etc. To life course theory, these shared circumstances and roles alter an historical event or context’s impacts: birth cohorts (what Hareven called ‘age cohorts’ above) are uniquely affected by historical events by virtue of being subject to the same life stage-based opportunities, constraints, etc., as above, when they encountered them. In the context of rapid social change, these can vary widely between birth cohorts, and their experiences – and these experiences’ consequences – can vary significantly. For example, Hareven and Adams, (2012) found that two birth cohorts – those born 1910–1919, who came of age during the Great Depression, and those born 1920–1929, who did so during WWII – had very different attitudes to, and practices surrounding, the family (Hareven, 1994, p. 453).

Thus, according to life course theory’s ‘timing of lives’ principle, an historical event’s ‘period effects’ – which affect the population regardless of age – are filtered through, and take special resonance from, birth cohort status. The term ‘cohort effects’ refers to the life-long ‘collective properties’ generated by the impacts of discrete historical events and contexts on individuals of broadly the same age. For example, the COVID-19 pandemic of 2020–2022 affected all age groups (period effects) but also affected specific birth cohorts in different ways (cohort effects): e.g. schoolchildren’s and university students’ educational trajectories were disrupted, and older adults experienced higher mortality.

While most often associated with the birth cohort, life course researchers have expanded the term ‘cohort’ to include members of different birth cohorts experiencing the same critical transitions - changes in role or status – at the same historical moment. In Ryder’s (1985, pp. 72–73) words, cohorts can be ‘identified by common time of occurrence of any significant and enduring event in life history … the strategic focus for research in social change is the context under which each cohort is launched on its own path’. Here, it is a group’s concurrent entry into new institutions, contexts, or activities that defines a cohort, regardless of birth year. As Dannefer and Settersten (2010, p. 5) write, ‘A cohort is generally defined as a collection of individuals who enter a system at a common time or time interval’. While ‘in studies of individual development and ageing, the entry point is typically defined by the year of birth’ (*ibid*), this broader definition allows for cohort effects that occur independently of age, life course stage, or even generation. Just as ‘a generation might consist of several cohorts, each of whom has encountered different historical experiences that have affected its life course’ (Hareven, 1994, p. 441), technically, a cohort conceived in this way can include members of different generations. Consider, for example, members of different generations who are diagnosed with the same medical condition in the same treatment era.

Because historical contexts and events ‘hit differently’ at different points in the life course, expected life course trajectories (e.g., marriage to parenthood to career advancement to funded retirement) can be interrupted, with long-term consequences, including cumulative disadvantage. For example, economic downturns affect birth cohorts differently, with many older workers needing to delay retirement due to these downturns’ immediate (in the case of workers on the verge of retirement) or cumulative financial costs. Moreover, because lives are linked through interdependent networks, cohort effects radiate outwards to shape the lives of others. A loss of financial resources, as above, could limit the ability to support one’s family, with both immediate and long-term effects (e.g., an inability to pay university tuition fees could negatively affect children’s own trajectories).

While often confused, generations and cohorts differ in key ways. Generation is typically used as a kinship term, denoting ‘a single stage or degree in the natural line of descent’ (Alwin and McCammon, 2003, p. 25), or [in Mannheim, 1952/1928 formulation] to people with ‘a shared temporal location (i.e., generational site or birth cohort), shared historical location (i.e., generation as actuality – exposure to a common period or era), and finally a shared socio-cultural location (i.e., generational consciousness – or “entelechy”) (Gilleard and Higgs, 2002, p. 373). In contrast, a cohort is ‘defined by its interaction with the historical events that affect the subsequent life course development of that group’ (Hareven, 1994, p. 441), while lacking a consciousness similar to that of a generation (there is no ‘cohort consciousness’). Thus, while generation exists both analytically and in the consciousness of its members, cohort is a purely analytic designation designed to tease out O’Rand’s (1996, p. 230) ‘historic interplay among people’s lives, structural contexts, and social change’, as above. Life course theory’s freedom to explore the impacts of historical events on lives over time among groups who are unaware of their cohort status widens its scope beyond that afforded by studies of generations: ‘The life course perspective seeks to make visible the significance of ‘macro’, or ‘distal’ social forces, including the social institutions and cultural practices that organize everyday routines, and unique historical events and periods of social change’ (Dannefer and Settersten, 2010, p. 4).

Moreover, members of specific birth cohorts may also be members of specific communities or groups whose distinctive experiences, circumstances, resources, and opportunities modify wider cohort effects, thus reflecting ‘intra-cohort differentiation’ (Dannefer, 1987). They would thus be members of ‘subcohorts,’ or subgroups within cohorts, distinguished by such important characteristics as racially minoritized status and class (Uhlenberg, 1996). For example, the Vietnam War affected African-American and/or lower-income American men of draft age far differently than it did their white and/or higher-income peers, as the former were far more likely to be drafted.

Cohort effects can be broadly divided into (a) perspectives and identities and (b) concrete or structural circumstances. Whittier (1997, p. 762), for example, found ‘striking’ cohort differences between activists who entered social movements ‘during separate cycles of protest’. As Whittier (1997, p. 763) writes: ‘When changes occur in the contexts that shape cohorts’ collective identities, recruits who enter movements at different times have different politicizing experiences and hence construct different collective identities’. As an example of structural opportunities and constraints, Borjas (2015) analyzed US census data from 1970–2010 to track immigrant earnings in the US and found clear cohort effects on entry wages and growth of earnings based on historical period of entry (pre- or post-1980s) into the US. More recently-arrived cohorts had ‘relatively lower entry wages (through 1990)’ and ‘a smaller rate of economic assimilation’ (‘the rate of wage convergence between immigrants and natives’) than did those who arrived before 1980 (Borjas 2015: 483-484). Moreover, ‘the evidence suggests that there has not been any economic assimilation for the cohorts that entered the country in the 1990s’ (*ibid*: 484), demonstrating cohort membership’s long-term consequences and role in driving cumulative disadvantage.

To life course theory, cohorts are not immediately evident in ways that other social commonalities are (for example, geographical region, nationality, age, race/ethnicity, and generation). As a result, applying the term ‘cohort’ based on birth year (but see below) is only meaningful if the historical period in which this group was born generated unique cohort effects (see, e.g., Settersten 1999, p. 121). Similarly, the ‘subcohort’ is a purely analytical designation based on the supposition that a particular characteristic alters an event’s cohort effects. In short, the existence of cohorts and subcohorts must be demonstrated. Identifying one group, uniquely positioned in both the life course and in history, as a cohort or, indeed, a subcohort, is a theoretical starting point, but only clear cohort effects can prove the existence of these groups as cohorts or subcohorts.

### 2.1 Queering the life course

While such critical concepts as cohorts, transitions, trajectories, and social change can help us uncover and document the complex realities of gay male aging, life course theorists have traditionally assumed heterosexual populations when considering what constitutes a critical transition and of what ‘the’ life course and its constituent stages consist. This critique has come from queer gerontology, one of queer theory’s (Seidman, 1994) most recent offshoots. As Sandberg and King (2022) note, in addition to problematizing aging studies for their heteronormativity (where ‘heterosexuality is viewed as the taken-for-granted and desirable norm’ – Sandberg and King, 2022, p. 1), life course theory relies on ‘heteronormative temporalities’ that follow the typical heterosexual progression from marriage to reproduction (Goltz, 2010; Ramirez-Valles, 2016). Similarly, Brown (2009: 76-77) highlights life course theory’s failure to consider ‘non-heteronormative models of families, social networks and intimate relationships’ on the one hand, and such important life transitions and trajectories as privately and publicly identifying as lesbian or gay, on the other – queer versions of the transitions and trajectories that are key to life course theory’s approach to aging and older age.

Because identifying as gay can occur at any age, gay men’s generational affiliations and identities may not neatly overlap with those of their heterosexual peers: Bitterman and Hess (2021) argue that gay men and women people may identify with two generations – that based on time of birth, and that based on when they identified as lesbian or gay and formed ‘a personal identity as an LGBTQ+ minority’ (*ibid*: 308). Moreover, the historically-contingent personal, social, and community relationships that lesbians and gay men form likely vary more widely and come into play at later points in the life course than they do among heterosexuals, whose sexual identities are typically formed in late childhood and young adulthood, and typically (although not always) remain the same over subsequent years. As Miller (2023, p. 2) recently wrote, ‘LGB [lesbian, gay, and bisexual] people have not historically met heterosexual-centric markers of adulthood (i.e., marriage, childrearing) and, in other cases, these major life events were delayed until fairly recently’. Thus, ‘the’ life course plays out differently in gay contexts, enmeshed as it is in ‘queer temporalities, where cultural narratives provide for alternative articulations of life courses, futures, and ageing, away from “straight time”’ (Sandberg and King, 2022: 4).

## 3 Lesbian and gay aging research

The earliest studies of gay aging, conducted in the 1970s (see, e.g., Kelly, 1977; Kimmel, 1978; Friend, 1980) necessarily relied on interviews gay men and women who had come of age well before the gay liberationist movements of the late 1960s/early 1970s. Pre-gay liberation, same-sex activities and relations were criminalized and medicalized and ‘passing’ as heterosexual was the most common way that gay men and women tried to avoid discrimination, rejection, and micro-aggressions. In the 1980s and 1990s, research brought inter-generational tensions – between gay men and women who came of age before and those who did so after the gay liberationist mandate to come out had become the dominant discourse within the gay community – to the fore. Grube, 1990, Grube termed these generations ‘natives’ (pre-gay liberation) and colonizing ‘settlers’ (post-gay liberation). But, while Whisman (1996) found that the men and women who identified as gay before gay liberation saw their homosexuality as an innate condition, and those who did so did so after saw it as a choice, it was not until the late 1990s that the cohort was used in studies of lesbian and gay aging research (see Stein, 1997).

Rosenfeld (1999) interviews with gay men and women born pre-1930 showed that differences in how they understood and managed their homosexuality (e.g., passing as heterosexual or proclaiming their identify as lesbian or gay) were linked to the era in which they identified as gay. Lesbian and gay identities developed during the pre- or the post-gay liberation eras produced very different understands of same-sex desire, self and identity, and relationships with gay and heterosexual people, across the life course and into older age. Rosenfeld coined the term ‘identity cohort’, comprised of individuals who identified as a member of a specific group during the same historical period in which a particular discourse (here, of same-sex desire and relations) was dominant. In her study, each identity cohort’s understandings of what it meant to be gay launched its members on a distinctive trajectory, as entering lesbian and gay networks and relationships at particular points in the gay community’s history steered them to new, and historically-specific, sexual identities, social connections and support networks, sexual and romantic practices, and opportunities and/or constraints.

Clearly, then, the historical era in which gay identities are formed has significant consequences for older age, and the evolution of these identities in historical context is a growing area of research. However, most gay aging researchers who consider historical context explicitly focus on generations (Lyons et al., 2015; Ramirez-Valles, 2016; Halkitis, 2019; Fredriksen-Goldsen et al., 2023), with some (e.g., Hammack and Cohler, 2011; Hammack et al., 2019; Bitterman and Hess, 2021) problematically using the terms generation and cohort interchangeably, and most trace historical factors shaping sexual identity development (see, e.g., Hammack et al., 2018; Bishop et al., 2020) rather than such concrete later-life circumstances as income. Far fewer have used the life course perspective to consider older gay men’s concrete circumstances or to explore cumulative disadvantage in these older populations (but see, e.g., Westwood et al., 2020; Miller, 2023).

Applying the life course approach to gay aging studies demands close attention to the historicity of the aging experience and of key life events: when in the life course gay men encountered specific historical contexts and events of direct relevance to them and to their futures and older age. This begs the question of which instances of social change were sufficiently significant to mark a division between cohorts. In the following section, we consider some of the historical shifts that, if considered through the lens of life course theory, could suggest distinctive cohorts of older gay men.

## 4 Dividing lines: lesbian and gay generations and cohorts

Given the many social changes that have occurred since the late 19th century of direct relevance to gay men – and, typically, that target gay people specifically – identifying key eras in gay men’s history is challenging, as many events or milestones overlap and coalesce within specific eras. Distinguishing between (a) discourses or ideologies of sexuality and same-sex relations (what some term ‘master narratives’ – see Cohler and Hammack, 2007) that affect self, identity, community, and socio-political responses, and (b) discrete socio-political events (e.g., the decriminalization and demedicalization of homosexuality) of direct relevance to the gay community, is not always self-evident, and different scholars have named LGBTQ generations differently. Drawing on the work of Halkitis, Cohler and Hammack, and others, Weststrate (2022, pp. 11–12) offered three ‘temporally bound narratives that describe unique cultural-historical epochs for LGBTQ+ self-making’, but we note that these epochs contain discrete events and sets of institutional practices that may demarcate historical dividing lines – before and after moments – and to suggest hitherto unconsidered gay male cohorts (a point we further explore below).

The first master narrative Westrate describes is the *narrative of silence* (pre-1960s), where what Rosenfeld (1999, 2003) called the stigmatizing discourse of homosexuality dominated, with LGBT people routinely harassed by police, discriminated against by employers, mistreated by a hostile medical and mental health enterprise as mentally ill, and prosecuted, as same-sex sexual encounters (and meeting places) were criminalized. Gay men, and heterosexuals, had very limited access to positive depictions of same-sex relations, identities, or encounters: as Rosenfeld (1999: 1) wrote, ‘Until gay liberation, riding the wave of the radical politics of the late 1960s, galvanized a nascent reformulation and representation of homosexuality, cultural depictions of homosexuality as anything other than a shameful, pathological condition leading to isolation and misery were censored and hard to come by outside certain (usually urban) areas’. The vast majority of gay men passed as heterosexual in all but private same-sex settings, although these private worlds have been mischaracterized as less rich and diverse, and as providing less solidarity and a weaker sense of community, than was actually the case (see Chauncey, 1994), and many large cities saw a massive growth of gay communities in the post-WWII years (D’Emilio, 1983). An incipient gay rights movement gained significant traction in 1969, when the Stonewall uprising, commonly considered the birth of the gay liberation movement, brought police harassment of gay people into the public eye.

Westrate’s *narrative of struggle and success* held sway throughout the 1970s and 1980s, when gay culture became much more visible and wedded to the gay liberationist mandate to ‘come out of the closet’ as both a political move and an act of self-empowerment. Gay communities worked to increase their political power and public recognition (through, for example, running for public office, organizing gay pride marches). The American Psychiatric Association’s removal of homosexuality from its list of psychiatric disorders in 1973 further galvanized gay activists dedicated to destigmatizing homosexuality and to freeing gay people from institutional control. This growing activism helped gay men to agitate for better responses to the AIDS crisis, which emerged in 1981 and decimated the gay male population until effective medications became available in 1996 (see below).

The third ‘master narrative’ described by Westrate is that of *emancipation* (1990s to the present day). Westrate notes several events that advanced the gay community’s rights and circumstances. The advent of effective anti-retroviral treatments (ART) changed HIV from a death sentence to a manageable condition with near-normal life expectancy (a theme we explore more fully below), the internet introduced new opportunities for gay men to connect outside of major urban centers, and ‘the 2000s ushered in an era of legal victories’ (*ibid*: 14) – for example, the US Supreme Court’s 2003 ruling that sodomy laws were unconstitutional, the 2015 passage of the Marriage Equality Act allowing same-sex couple to legally marry, and the 2011 repeal of Don’t Ask, Don’t Tell, which had allowed gay people to serve in the US armed forces only if they kept their sexual identities secret.

Westrate terms those who came of age at the time any one of these narratives was dominant the *invisible generation* (pre-1960s, narrative of silence), the *Stonewall generation* (1970s-1980s, narrative of struggle and success), the *AIDS generation* (which Halkitis, 2013 – argues contained those who came out in the 1980s and 1990s, and which spanned the narrative of struggle and success and the narrative of emancipation), and the *Queer generation* (the narrative of emancipation).

As noted above, most research linking the lived experience of older gay men to historical contexts and events explores generational impacts rather than cohort effects, even though the increasingly rapid changes described above could easily constitute dividing lines between distinctive cohorts of gay men. While these broad changes contain a raft of discrete ‘cultural-historical events’ (Weststrate and McLean 2022) that could (and no doubt did) shape gay men’s identities, opportunities, and constraints at different points in the life course, in the following section we focus on events with potential cohort effects generating cumulative consequences for gay men’s concrete later-life social and economic circumstances. To reiterate, as cohorts are analytic designations, we cannot state unequivocally that these events will generate cohort effects – with the exception of some proven cohort effects within the AIDS generation, as below, future research would need to establish clear cohort effects to justify declaring those who experienced the same critical transition before and after key events to be members of different cohorts.

## 5 Cohorts and cohort effects among older gay men

Between 1981 and 1996, when ART first became available, HIV almost invariably progressed to AIDS, a fatal condition of severe immunocompromise that allowed opportunistic infections to take hold and progress unchecked (Trickey et al., 2024).

Before HIV spread to other countries and populations, AIDS and AIDS deaths were most densely concentrated in the gay male population in the West, sparking anti-gay, and anti-sex, reactions from state and federal governments. In the US, AIDS primarily killed gay men living in ten major cities with thriving gay communities (Rosenfeld et al., 2012), directly affecting tightly knit gay male friendship and community networks and intensifying AIDS’ personal and community-level impacts on gay men. According to Gagnon and Nardi (1997), by 1995, one in ten gay men in the US aged 25–44 had died due to AIDS. Many gay male members of the AIDS generation lost multiple friends and/or partners at the epidemic’s height, with smaller social networks in later life as a result (many in their later-life networks, also living with HIV, can offer only limited support due to their own ill health). Moreover, many alive today had themselves acquired the virus (and even survived AIDS) pre-1996. These experienced poor health before ART became available and are now living with the long-term effects of this poor health and of HIV itself: older people living with HIV have higher rates of comorbidities, multimorbidity, functional decline (and of such conditions as cardiometabolic disease, renal and liver disease, certain malignancies, cognitive decline, and osteoporosis, at younger ages; Crane and Drumright, 2022) and, often, of early medications’ side effects. They also experienced fearing their own death and ineffective medication regimens that caused significant (and often long-lasting) side-effects.

The AIDS epidemic emerged during Reagan’s presidency, when leaders of the Christian right ‘contended that abortion, feminism, and homosexuality represented a multifaceted “attack” on the family’ (Dowland, 2009, p. 607), depicting gay men as endangering children by normalizing homosexuality and being more prone to abusing children than were heterosexuals (*ibid*: 626). At the same time, ‘big government’ and the welfare system were depicted, primarily by the Reagan administration, as obstacles to economic prosperity and personal responsibility (O’Connor, 1998). In the context of right-wing attacks on sexual minorities, and with growing fears over immigration, President Reagan (‘notably silent on the issue of the growing AIDS crisis’ – Travis, 2022, p. 18), ‘pressured to demonstrate efforts to combat the HIV/AIDS epidemic … required all immigrants be tested for HIV, and that HIV infection (with or without AIDS) be included as a disease of public health significance. This change was quickly passed by Congress, making all aliens infected with HIV ineligible for admission to the United States’ (Winston and Beckwith, 2011, p. 709). Gay communities responded to the AIDS crisis, government indifference, and right-wing, often evangelical hostility by caring for people with AIDS and forming activist groups such as ACTUP (the AIDS Coalition to Unleash Power). These groups agitated for faster, more effective scientific responses, increased government HIV/AIDS budgets, better access to treatment and services, and an end to proposals for such oppressive measures as quarantining people with HIV/AIDS (Stockdill, 2013).

In the epidemic’s early (1981–1996) years, many who had been diagnosed with HIV or AIDS left employment due to ‘the inflexibility of adapting work hours due to physical limitations, or increasing fears of being discovered and being subject to discrimination in the workplace’ (Bourgeois, 1998, p. 105) or to being advised to do so and to liquidate their assets to ‘take advantage’ of the ‘years they had left’. Once these men’s health improved due to ART, many could not find work after many years of being out of the workforce. Thus, while, as Bourgeois (1998, p. 115) wrote of her sample of gay men with AIDS aged 22–44, interviewed in the 1990s, ‘the resolution of adult developmental tasks – attaining independence from family, developing a fulfilling career, establishing intimate relationships, attaining financial independence, and maintaining optimum physical health – was dramatically interrupted by AIDS’, HIV and AIDS had more long-term effects, driving cumulative disadvantage into later life.

While the AIDS epidemic affected virtually all gay men who lived through it, the Baby Boom (born 1946–1964) generation was hardest hit, both by virtue of the sheer number of deaths and by witnessing the toll that these deaths took on their immediate communities. A significant body of research has explored the various social, health, and mental health impacts of the AIDS epidemic on older gay men (e.g., Stulberg and Smith, 1988; Martin and Dean, 1993; Cherney and Verhey, 1996; Rosenfeld et al., 2018a) – although, as ART significantly lowered mortality rates, this has changed to a concern with the impacts of HIV – and some have considered the ways in which they coped with the epidemic in all its forms both at the epidemic’s height and in older age (see, e.g., Machado, 2012; Halkitis, 2013; Rosenfeld et al., 2018b; Sichel, 2022). However, as Tester (2018) notes, most research in this area focuses on men who were already enmeshed in the gay community when the epidemic first struck.

### 5.1 HIV/AIDS, cohorts, and subcohorts

AIDS deaths were not uniformly distributed across the AIDS generation: as we can see above, we can broadly divide this generation into two main cohorts – those diagnosed between 1981 to 1996, and those diagnosed post-1996. But within the 1981–1996 cohort, other notable differences emerge. As Rosenfeld’s analysis of Centers for Disease Control 1987–1997 data (Rosenfeld et al., 2012) showed, while men aged 25–44 in that decade accounted for 72% of all AIDS-related male mortality each year, men aged 35–44 experienced the highest mortality rates of all age groups across all 10 years of data: ‘AIDS deaths among men aged 35–44 years [born 1951–1960] remained highest within this birth cohort even when they consisted of those who had matured from the relatively protected age group of 25–34 years into this older group … This, then, was the age group around which AIDS deaths increasingly clustered’ (*ibid*: 259). The concentration of 1987–1997 gay male AIDS deaths in close-knit gay communities in a small number of cities became even more exaggerated by their concentration within a specific age group and a small number of successive birth cohorts and by taking place over a single decade. Moreover, ‘within the 25–44 age grouping, a higher rate of deaths per 100,000 occurred among African American … men than among White men; in 1995, this was 106.3/100,000 versus 23.9/100,000, respectively’. African-American men ‘aged 34–44 years account[ed] for 69% of the AIDS deaths in their cohort in 1995, 67% in 1996, and 65% in 1997’ (*ibid*: 258–259).

Building on Rosenfeld’s work, Tester identified three subcohorts of gay men who lived through the epidemic’s early years (1981–1996), each of whom experienced the AIDS epidemic very differently based on the historical era in which they entered and forged connections within urban gay communities. The *entrenched* cohort had strong connections to the gay community pre-1981; its members cared for and lost many friends and partners to AIDS and felt the impacts of AIDS at a deeply personal and at a wider community level. Gay men who had only come out as gay in the late 1980s or early 1990s or had come out earlier but were less connected to gay men, often because they lived outside of major cities, were relatively *insulated* from the devastation the AIDS epidemic wreaked on the gay community – the men they saw die of AIDS were typically not close friends or lovers. Men who came out ‘and began strongly connecting with gay men in urban communities around or after 1996’, when AIDS deaths began to drop, were *removed* from the worst of the AIDS deaths and the toll they took; ‘they experienced no personal loss and little to no community loss’ (*ibid*: 40). Connecting to urban gay communities as gay men at different periods in the history of HIV/AIDS shaped these men’s experiences of personal and community loss and support.

### 5.2 Post-1996 cohorts of older gay men living with HIV

While demonstrating the importance of considering both birth cohort and subcohort membership when investigating gay men’s later lives, both Rosenfeld et al.’s and Tester’s research focuses exclusively on the pre-1996 context. We suggest that this does not fully reflect all of the dividing lines within the ongoing history of the HIV/AIDS epidemic that could signify distinct cohorts. Here, we refer to periods of change in ART post-1996 whose potential role in generating cohort effects has not yet been considered by researchers exploring the impacts of HIV on older gay men.

Given how critical HIV medications are to people living with the virus, changes to medical treatments spark significant changes in the subjective meaning and management of the diagnosis. Catalan et al. (2024, p. 5) found that ‘the psychological impact and mental health problems associated with HIV changed over the decades in parallel with the medical and therapeutic progress that took place during that time’, clearly linking changes in experience and outlook to changes in medication and treatment. Before 2009, ART was only prescribed after HIV had progressed. ART regimens during those years were often complex and could cause significant side-effects. By 2010, ART had become ‘easier to take (one tablet a day) and had fewer side effects’ (Alcorn and Pebody, 2023), adding better quality of life to a treatment regime that had offered increased longevity at the expense of the challenges described above.

Moreover, the advent of HIV prevention through ARTs has changed sexual practices by allowing gay men and others to have condomless sex without acquiring HIV. Pre-exposure prophylaxis, or PrEP, ‘is a daily course of tenofovir (also known by its brand name Truvada) that has a significant protective quality after 5 days against HIV acquisition—up to 92% with medical adherence’ (Spieldenner, 2016, p. 1686). Thus, people living with HIV who are on ART and have an undetectable viral load are unable to transmit the virus. In the US, PrEP was first approved for HIV prevention in 2012. PreP has had a direct influence on gay men’s social networks and interactions, as they re-gained agency (and freedom) to engage in and maintain sexual and romantic relationships (Montes, 2020) with almost no risk of transmitting HIV through sex. As Montes argues (2020), PrEP likely reduced stigma and anxiety and increased trust among gay men and their partners, thus potentially strengthening partnerships and encouraging their longevity. Engaging in sexual and romantic relations with other men in an era when the HIV prevention message that ‘U = U’ (Undetectable is Untransmittable) is widely known and understood by the gay male community may have broken down some of the barriers to intimacy between men living with HIV and men who are not.

However, generational and cohort differences, and intracohort variation, are evident here as well. Inadequate distribution and coverage of PrEP exist across and within countries (Bavinton and Grulich, 2021), so that white, middle- and upper- class gay men living in urban areas have the most access to PreP, directing us, again, to issues of intra-cohort variation and cumulative dis/advantage. Recent research (Holt et al., 2023) has found that, while overall HIV prevention coverage (using one or more means of prevention) was highest in men aged 45 and above. PrEP use was concentrated among gay men aged 25–44, with older gay men using ‘the most varied mix of prevention methods, employing PrEP, condoms and undetectable viral load, or avoiding anal intercourse with casual partners’ (*ibid*: 12). Being diagnosed with HIV in one or the other of these periods – 1997-2009, 2010 to now, and post 2012 – introduced distinctive practices and concerns that could very well have shaped gay men’s HIV-related experiences and expectations, both at the time and into later years. Hammack et al. (2019) found that gay men’s views of PreP differed by generation, with men born in the 1950s-1960s (AIDS-1 Generation) and in the 1990s (the Post-AIDS Generation) viewing PreP more positively than did those in the middle generation (the AIDS-2 Generation, born in the 1970s-early 1980s). The authors linked these differences to how each generation was positioned in relation to the AIDS epidemic’ and to the dominant practices and concerns in gay men’s sexual culture. Thus, as in Tester’s work (above), the historical time of entry into the gay male world shaped attitudes towards a new HIV prevention technique.

### 5.3 The Don’t Ask, Don’t Tell policy

The Don’t Ask/Don’t Tell policy was adopted in 1994 in the US, officially barring individuals from serving in the military as openly LGBTQ people. People found to be LGBTQ were less-than-honorably (or other-than-honorably) discharged. Same-sex behavior in the military was prohibited before the 1994 policy (‘over the last 70 years, an estimated 100,000 military veterans either left or were kicked out of the service for their sexual orientation’ – Franklin, 2021), but Don’t Ask, Don’t Tell, proposed as a political compromise between those wanting LGBTQ people to be able to serve openly and those wanting them excluded or removed from military service altogether, allowed LGBTQ people to serve, but only, again, if their sexual orientation was kept private (Alford and Lee, 2016). According to the Service Members Legal Defense Network (2011), under Don’t Ask, Don’t Tell, over 14,000 service members were discharged between 1994 and 2011.

These restrictions were a source of severe stress for lesbian and gay service personnel, and the lack of privacy while on active duty, and the military’s right to tap their telephone calls and open their mail, made fears of being discovered to be lesbian or gay even more severe. A survey of LGBT veterans found that ‘36% were investigated for their sexual orientation, 15% reported isolation due to sexual orientation, 11% were forced to participate in a psychiatric evaluation related to their sexual orientation, and 2% were incarcerated for their sexual orientation’ (Ramirez and Bloeser, 2009, p. 14).

Being less-than-honorably discharged meant losing important benefits, such as health care, home loans, and tuition for education, and, assuming gaps in employment post-discharge, lost salary and lost pension contributions. Writing in 2007, Westcott and Sawyer, (2007) noted Don’t Ask, Don’t Tell effective barring of lesbian and gay service personnel from eligibility for medical benefits for partners and/or children, supplemental housing resources, and survivor benefits. The authors note that all service members must enroll in the Defense Enrollment Eligibility Reporting System, through which they must declare any children and/or spouse, with failure to do so a breach of the Uniform Code of Military Justice. While their heterosexual counterparts could do this without fear and in the knowledge that they would receive these benefits, for gay service men and women, ‘The forms required to “prove” that a child is the dependent of a service member and the paperwork required to ensure care for the child when a service member is deployed or otherwise unable to care for the child raises the risk of revealing a “Don’t Ask, Don’t Tell” violation’ (*ibid*: 1124–1,125). As a result, ‘gay service members who want to protect their loved ones or same-sex partners face significant risks under “Don’t Ask, Don’t Tell” if they choose to apply for these benefits’(*ibid*: 1127), and ‘introducing children into a same-sex relationship setting can drastically raise the risk that a gay service member’s sexual orientation will be discovered and the service member will be fired’ (*ibid*: 1134).

After the law was repealed by President Obama in 2010, those discharged under Don’t Ask, Don’t Tell were allowed to re-enlist, and, in 2021, ‘LGBTQ veterans who were discharged from the military under the “Don’t Ask, Don’t Tell” policy … gained new access to full government benefits from the Department of Veterans Affairs’ (Franklin, 2021). Gay men discharged under Don’t Ask, Don’t Tell could apply for a discharge upgrade or correction which, if successful, would make them eligible for benefits earned during their service (Veterans Affairs, n.d.,). Three years after the repeal, Geidner (2013) wrote ‘people discharged from the military under “Don’t Ask, Don’t Tell” since November 10, 2004 who had only received one-half separation pay following their discharge but who otherwise would have received full pay now will be entitled to that full separation pay’.

### 5.4 Marriage equality

In 2013, the US Supreme Court overturned the 1996 Defense of Marriage Act (DOMA). DOMA had banned federal recognition of same-sex marriages, although these had already been recognized by a number of US states, and some localities allowed same-sex couples to be legally recognized through civil unions and domestic partnerships. In 2015, the US Supreme Court ruled that same-sex couples had the same rights to marry as did heterosexual couples, granting them associated benefits such as spousal inheritance (without penalties), access to a spouse’s employers’ sponsored health insurance, medical decision making and visitation rights for spouses, and a fair separation of resources post-divorce.

These rulings had direct financial impacts on gay men. Writing in 2000–13 years before the Marriage Equality Act passed – Cahill et al. (2000) noted that only ‘married spouses are eligible for Social Security spousal benefits, which can allow them to earn half their spouse’s Social Security benefit if it is larger than their own’, and while ‘Medicaid regulations protect the assets and homes of married spouses when the other spouse enters a nursing home or long-term care facility’, these benefits were not available to same-sex partners (Cahill et al., 2000, p. 2). That US tax law and pension policies did not grant same-sex partners the same benefits as they did to married couples cost ‘the surviving partner in a same-sex relationships tens of thousands of dollars a year, and possibly over $1 million during the course of a lifetime’ (*ibid*). As a consequence, same-sex couples were financially disadvantaged relative to their heterosexual peers, earning, on average, 34.7% less in retirement income (Bennett and Gates, 2004).

Marriage equality also offered other financial advantages: as Badgett et al. (2021, p. 158) summarized, Sansone (2019) ‘found evidence that marriage equality significantly increased probabilities of being employed among individuals in same-sex couples’, and ‘argued that a key mechanism was reduction in discrimination against sexual minorities’, potentially linked to wider cultural changes that the Marriage Equality Act triggered, but also possibly due to the state in which same-sex couples live. Badgett et al. cite several studies that found that legal, federally recognized same-sex marriage increased savings and home ownership, ‘was associated with significant increases in applications for mortgage credit’, and ‘increased health insurance coverage and access to care for men in same-sex households’ (*ibid*).

Clearly, the passage of the Marriage Equality Act could neither erase nor compensate for previous years, even decades, of same-sex couples’ lost income, but it did offer older gay men opportunities to begin to recoup some of these losses and offered younger cohorts of gay men and women the same financial basis on which to begin married life. Because years of lost income and denial of benefits directly impact later life (consider pension income, and Cahill et al.’s reference to Medicaid above), and have cumulative consequences, long-term same-sex romantic relationships formed before or after the passage of the Marriage Equality Act would have had very different legal and financial implications, launching same-sex partners on different paths. Nonetheless, the Marriage Equality Act’s impacts will be neither immediate nor unaffected by enduring patterns of anti-gay discrimination: as Jepsen and Jepsen (2020, p. 16) found after analyzing 2001–2018 data on cohabiting couples in the US, the financial ‘penalty for gay males relative to married men’ has endured: ‘the gap in wages by sexual orientation narrows between 2001 and 2008. After that, the gap remains relatively flat for gay men at around 11 percent for annual wages, earnings, and income’.

Similarly, being allowed to re-enlist in 2010, and gaining new access to full government benefits from the Department of Veterans Affairs in 2021, gave gay men and women who had been discharged from the military between 1994 and 2010 under Don’t Ask, Don’t Tell access to important and financial health care benefits. While these strengthened their ability to care for themselves, their families, and their later-life financial health, the actual costs to these in the intervening years have yet to be established. Thus, while the mental toll that this interruption to their expected trajectories is becoming known, the cohort effects with which we are concerned here (social and economic status) remain unclear.

## 6 Future cohorts, future research

We can research these three instances of social change – developments in HIV treatment, the Don’t Ask, Don’t Tell policy and its revocation, and the Marriage Equality Act – now, as an older gay male population will likely be (or likely has been) affected by these changes, either in later life or in the years leading up to older age. Gay men aged 65 and older (born 1959 or earlier) would have witnessed the Marriage Equality Act (2013) aged 54 or older, Don’t Ask, Don’t Tell (2010) and early access to simpler, less disruptive forms of ART (2010) aged 51 or older, and PreP (2012) aged 53 or older. Following our understanding of cohorts as comprising both those (a) born in the same year or years and (b) entering into new institutions, contexts, or activities at the same historical moment, regardless of birth year, we can compare older gay men who entered into long-term partnerships, the US military, and new HIV treatments and prevention technologies before and after these events occurred to determine if these changes, and suggested cohort memberships, generated real cohort effects (measured by a range of outcomes, including identity, social relations and support, income, physical and/or mental health, and outlook). While these events only occurred in the last 15 years, limiting our ability to isolate cumulative advantages and disadvantages to the degree that earlier events permit, they nevertheless provide opportunities to test the real existence of these suggested cohorts, and to begin to track related cohort effects over time should preliminary findings provide concrete evidence that these cohort designations are more than analytic conceits.

Other significant social changes, however, do not allow for current investigation into long-term cohort effects on older gay men. This is because they have happened so recently that cohort effects would be difficult to identify, these changes’ impacts will not yet have taken root, and we do not yet know if these legislative changes will be significantly altered (at the time of writing this article, the US is poised to elect a new President, and the outcome may reverse some key decisions and policies that grant LGBT people equal, or close to equal, rights). However, we can identify some recent social changes of direct immediate and long-term relevance for gay men across the life course, and which we suggest could, theoretically, have cumulative consequences, and thus shape a different old age for future cohorts of gay men. One such change is the 2020 anti-discrimination legislation, which we consider below. By the same token, we cannot yet (and will not be able to do for another 40 years) identify long-term cohort effects among the queer generation, now aged, at the oldest, 24. However, given this generation’s distinctiveness, and the social changes to LGBTQ+ people that its members have already experienced, we consider it worth exploring (a) the unique perspectives and experiences that the queer generation will bring to its maturation and later life, and that will shape how they respond to future such changes, and (b) recent and current events that could already be directing them along certain paths.

### 6.1 Anti-discrimination legislation

In 2020, in Bostock v. Clayton County, the US Supreme Court ruled that the protections against discrimination in hiring or employment on the basis of ‘race, color, religion, sex, or national origin’, afforded by Title VII of the 1964 Civil Rights Act of 1964, apply equally to sexuality and gender identity, as these are covered by the term ‘sex’ in the original Act. While attempts had been made, sometimes successfully, to ban anti-LGBT employment discrimination, this was the first federal decision that affected both government and private workplaces (as a federal law, it applied to all 50 US states and the District of Columbia). Although it is too soon to assess this decision’s impacts on gay men’s life-long earnings and financial resources (including work-place provided health insurance, pension plans, etc.), it is clear that this legislation constitutes a critical historical moment suggesting eventual cohort effects, given the overwhelming evidence of employment discrimination against gay men and women (see Badgett et al., 2021). Protection from employment discrimination, itself of significant immediate and long-term financial advantages, further catalyzes the Marriage Equality Act’s financial benefits: equal access to employment-based spousal benefits (e.g., sharing work-provided health insurance) is meaningless if neither same-sex married partners is hired.

The potential impacts of entering the labor force before or after this decision was made, or of experiencing this legislation at different points in the life course, are clear: the stress and disadvantage that gay men undergo when faced with, or fearing being faced with, anti-gay discrimination in hiring, promotion, and/or workplace treatment will likely be, at the very least, lessened post-2020, with lesbians and gay men now able to contest discriminatory treatment in court. Removing barriers to hiring, promotion, etc. will increase their financial health and security, with cumulative consequences for later-life (e.g., pension income, savings, home ownership, and affording health and long-term care). These will also benefit gay men’s children and grandchildren, introducing cohort effects to subsequent generations.

### 6.2 The queer generation

The queer generation (born late 1990s-early 2010), came of age under what Weststrate (2022) called ‘the narrative of emancipation’. Now in its teens to early adulthood, this generation uses social media to connect to other LGBTQ people and rejects binary gender identities and the categories of gay, lesbian, bisexual, and transgender, embracing instead a queer collective identity centered on gender and sexual fluidity. Members of this generation adopted sexual and gender identities (including gender transition) at much younger ages than had previous generations (Russell and Fish, 2019).

However, while Weststrate (2022) considered the ‘narrative of emancipation’ to continue to today, this narrative is being strongly contested. While coming of age – and political awareness – in the relatively tolerant Obama era, the queer generation is now experiencing a severe backlash from right-wing politicians, citizens, and media. These have called for, *inter alia*, legislation to constrain (or eliminate) gender transition procedures, LGBTQ+ literature and instruction, and non-binary use of restrooms and participation in sports. While much of this backlash focuses on elementary and high schools (Kindy, 2022), ‘the deleterious effects of these legislative efforts are seeping into higher education, normalizing antagonism toward LBGTQ+ students on some campuses and creating additional pain and stress for a population that already bears more than its fair share’ (Carrasco, 2022). Thus, this generation is, at an early and formative age, being targeted by anti-gay/anti-queer forces to a degree not seen in the US since the McCarthy era, while at the same time being highly conscious of its queer identities and its distinctive generational status, and uniquely connected to other queer people through the internet.

The potentially long-term impacts of these anti-LGBTQ+ measures on a self-conscious queer generation are already being felt. Many young queer people are not applying to colleges and universities in states that have passed, or are planning to pass, anti-LGBTQ+ laws (Horowitch, 2023), and parents of young transgender children are moving from states that have passed laws banning gender reassignment for children and/or discussion of LGBTQ+ issues in schools – a decision that could also be made by parents of cisgendered queer children seeking to protect these children from anti-LGBT treatment by institutions and others in their communities. In keeping with life course theory’s ‘timing of lives principle,’ these measures, and the politics that ground them, have already affected members of the queer generation differently than they have members of other generations and birth cohorts within the queer generation (for example, choosing colleges and universities to attend would have been a very different experience for those who have already graduated from college or university before these anti-transgender measures were passed or proposed than for those in high school at the time). Focusing on selection of colleges or universities, we can propose two subcohorts of queer teenagers: those who could and those who could not afford to choose which university to attend based on the host state’s politics surrounding LGBTQ+ rights. Other intra-cohort variations and cohort effects will declare themselves as this generation ages.

### 6.3 Intra-cohort variation among older gay men

As noted above, assuming a universal aging experience based on age alone obscures important differences within generations and within and across birth cohorts. That African-American men aged 35–44 in the 1987–1997 decade had AIDS mortality rates roughly four times as high as those of their white peers (Rosenfeld et al., 2012) both marks them as a subcohort of gay men their age killed by AIDS in those years and demonstrates the dangers of melding distinctive communities and groups into a single generation or cohort. The same point can be made in relation to the 2020 anti-discrimination act, which, while benefitting all gay men, would more severely affect African-American and Latinx gay men who disproportionately have faced barriers to better-paid employment and promotion due to institutional racism (Kum, 2017).

Life course theorists have emphasized the various ways that social statuses such as gender, racially minoritized status, and class (Fredriksen-Goldsen et al., 2014; Holman and Walker, 2021) can filter broader cohort effects to create subcohort effects, as racial, gender, and social class systems and even geography (see, e.g., Lewis, 2019) expose different minoritized groups to distinctive histories. For example, African-Americans’ experiences, outlooks, concerns, and circumstances are likely as affected by their unique history of racist oppression and community resistance as they are by heteronormative, and homophobic, contexts and events. Such monumental social changes as the Jim Crow laws, the great migration North, the Civil Rights and Black Power movements, Dr. Martin Luther King’s assassination, Obama’s election, and the Black Lives Matter movement have shaped their socio-political landscape and consciousness more saliently than they have other groups. Contrary to the now-debunked myth that ‘racial/ethnic identities clash with LGB identities’ and that African-American LGBTQ people are ‘an outsider group with no or little commonalities with a supposedly mainstream—by which is usually meant White—LGB community’ (Meyer, 2010, p. 2), African-American gay men are also deeply connected to gay communities, with a rich history of gay life within the African-American community and deep historic connections to and involvement with the White gay movement. Thus, for older African American gay men, shifting, but enduring, configurations of oppression and exclusion have intersected with African-American activism and the instances of social change we have explored above, shaping identity, community relations, and later-life dis/advantage.

Similarly, that African-American and Latinx men and women are over-represented in military service (see, e.g., Barroso, 2019) generates disproportionate impacts of Don’t Ask, Don’t Tell on those groups of gay men. Writing in 2010, a year before Don’t Ask, Don’t Tell was repealed, Gates noted that while ‘The total number of discharges has been declining since 2001 when 1,227 men and women were discharged’ (Gates, 2010, p. 2) (in 2009, roughly one third that many service members were discharged under Don’t Ask, Don’t Tell), these declines were more pronounced among White (non-Latinx) service members than their African-American and Latinx counterparts: ‘The declines are somewhat more modest or not really evident among other racial and ethnic groups’ (*ibid*). As a result, ‘women and racial/ethnic minorities now bear a larger portion of the burden imposed by the policy than they did when the policy was first implemented in 1993’ (*ibid*: 3). While the number of racially minoritised men men discharged under the policy is small (1,570 African-American men between 1997 to 2008, and 1,022 Latinx men in the same period – *ibid.*: 3), the emotional and financial stress of being discharged, and of seeing such high proportions of other African-American and Latinx men being discharged, would have generated clear subcohort effects on these gay men – effects which, we have argued, would shape their later lives.

Likewise, there are subcohorts of older gay men people linked to the social change in immigration policy. In 2010, the US repealed the ban on people living with HIV immigrating to the country. As explored above, the 1987 ban resulted from the intersection of that era’s clearest biases: racism, homophobia, and the stigmatization of people living with HIV. This change altered the concrete realities of many gay men: in addition to lessening HIV-related stigma, many non-citizens living with HIV who immigrated to the US were men (and gay men – Winston and Beckwith, 2011). For some gay men from Latin America and Africa, the 1987 ban’s repeal was a literal lifesaver, as, like those who had immigrated to Canada, they became able to access HIV treatments unavailable in their countries of origin.

The Marriage Equality Act also spurred another significant wave of migration to the US among gay men, as it afforded gay married couples the same access to same visas as heterosexual married couples. In 2011, The Williams Institute (2011) estimated that approximately 40,000 binational and dual non-citizen same-sex couples living in the United States were discriminated against by being barred from securing the same immigration rights as heterosexual couples (most of these 40,000 same-sex were from Mexico). After the Marriage Equality Act, approximately 1.5 million mixed-citizenship same-sex couples legalized their marriages in the US (Redpath, 2022). Alongside differences in ethnicity and culture when compared to White, US-born gay men who married under the Marriage Equality Act, moving from their countries of origin to marry in the US, with all that doing so entails, suggests a sizable subcohort of gay men who legally entered into same-sex marriage.

### 6.4 Challenges in identifying cohort effects among older gay men

Although data sets that gather information on sexual orientation are becoming more common, we still lack robust, longitudinal data for identifying cohorts and cohort effects within the older gay male population (Black et al., 2000) – a lack of particular importance given that self-administered, online, anonymous surveys are the data collection methods that are ‘least likely to underestimate the prevalence of non-heterosexuals’ and ‘will produce more accurate estimates of non-heterosexual prevalence’ (Robertson et al., 2018, p. 1078). Surveys’ failure to include sexual orientation (see Sell and Holliday, 2014) has made it necessary to, for example, extrapolate the number and characteristics of same-sex couples from household composition – a technique that leaves substantial room for error. Similarly, analytically identifying a survey respondent as lesbian or gay based on number of past same-sex partners alone elides the complex dynamics that go into identifying as gay. Because we have only a small window of opportunity to explore cohort effects among gay men born in pre-WWII, adding sexual orientation and gender identity questions to current national panels, such as the Health and Retirement Study, is especially pressing, as is conducting smaller-scale (including qualitative) studies with diverse samples of gay men born pre-WWII (Ramirez-Valles, 2024).

However, some data sets that include sexual orientation are becoming available, although they only started doing so recently and so do not allow for longitudinal analysis that includes older gay men born in the pre-war years. Starting in 2013–2014, the National Health Interview Survey has included a question on sexual orientation. In 2018, the Centers for Medicare and Medicaid Services and the Office of the National Coordinator for Health Information Technology began requiring all electronic health records ‘certified for Meaningful Use’ to include sexual orientation and gender identity (Cahill et al., 2016, p. 100; see also Grasso et al., 2019). Many of these data sets focus on physical and mental health, drug use, and sexual behaviors, thus sidelining the socio-economic cohort effects with which we have been concerned here. However, more recently (2021), the US Census Bureau announced that its Household Pulse Survey – part of the American Community Survey – ‘will now ask respondents their sexual orientation and gender identity (SOGI) in addition to their sex’ (US Census Bureau, 2021). These and similar data sets will allow researchers to track gay men’s resources, social networks, employment, marital and parental (and grandparental) status, migration patterns, and physical/mental health, consider these outcomes and changes in circumstances in light of social changes of direct relevance to gay men (thus potentially identifying gay male cohorts), and uncover and document intra-cohort variations linked to, e.g., ethnicity and racially minoritized status (thus potentially identifying subcohorts).

## 7 Discussion

Most research investigating aging among gay men that is attentive to the interplay between individual lives, social change, and structural contexts focuses on the role of generational differences and dynamics in shaping gay men’s identities and outlooks, rather than on gay men’s later life concrete circumstances as cohort effects – products of gay men’s critical transitions unfolding within contexts of social change. We have argued that these generationally-focused studies yield important, but limited, insight into the factors shaping gay men’s lives over time, across the life course, and into older age – insights which life course theory is specifically designed to generate. Life course theory’s focus on cohorts, groups of people united by a common timing of entry into a system or context but without being conscious of their status as cohort members, affords it analytical access to ‘distal’ forces that intersect with instances of social change and individual to shape older age – an access that is less available to scholars focusing on generations. In the case of older gay men, such macro or distal forces that have and will continue to shape their lives include the class system, the racial system, the economy, the state; such wider cultural biases as racism, sexism, homophobia, anti-immigrant prejudice, and HIV-related stigmatization; and institutions (i.e., the banking and insurance industries) that perpetuate and/or are bounded by these forces. All of these distal forces, as we have shown, directly affect gay men’s social positions, experiences, opportunities, and constraints across the life course.

Life course theory can be especially useful for uncovering and documenting the realities of gay men’s aging and later life. The rapid, multiple, and ongoing changes in how same-sex relations and identities are understood and managed, both by gay men themselves and by other individuals and agencies, provide a wealth of instances of social change directly affecting gay men. It is the intersection of these events with individual lives and the timing of those lives that generates cohort effects – again, life-long ‘collective properties’ generated by the impacts of discrete historical events and contexts on individuals of broadly the same age, in the case of birth cohorts, or on those, in Ryder’s words, sharing a ‘common time of occurrence of any significant and enduring event in life history’ (see above).

In the case of gay men, both cohort types are highly apposite for older age. Being born in one era exposes us to widely different ways of understanding and experiencing same-sex relations, shaping our initial outlooks. The wide range of ages in which men identify as gay and enter into gay communities and relationships (clearly a ‘significant and enduring event in life history’, as above) connects them to others who underwent the same critical transition at the same time, although these other men may be older or younger and thus members of different birth cohorts (and, indeed, even generations). Thus, for example, AIDS deaths in the 1980s and 1990s clustered around a small number of birth cohorts, and men who entered into gay life at different points in the epidemic’s rapidly-unfolding history experienced both the epidemic and gay communities very differently.

The sheer number of social changes that have occurred in the post-WWII era (*inter alia*, the homosexuality’s demedicalization and decriminalization, the emergence of gay liberation, the AIDS crisis, and life-saving HIV treatments, the passage of key legislation directly affecting gay men), intersecting with critical life transitions, suggests that multiple gay male cohorts, and multiple cohort effects, are woven into the gay male community’s fabric. But these changes and cohort effects also likely appear within the same gay male life – consider a gay man who was diagnosed with HIV, entered the military, and formed a life-long partnership before or after each of the events we have explored above took place. This is further complicated by the fact that cohort effects are modified by such statuses as gender, class, racially minoritized status, age, citizenship, etc. For example, as we have briefly explored above, instances of social change intersect with racially minoritized groups’ distinctive histories and circumstances (consider the disproportionate impacts of Don’t Ask, Don’t Tell on African-American and Latinx gay servicemen, of the repeal of the ban on people with HIV immigrating to the US on gay men from Latin America and Africa, and of the Marriage Equality Act on non-US citizens).

Gay men may therefore embody multiple cohort memberships and cohort effects, and how these intersect within the same life to shape older age is a challenge that we suggest gay aging studies are best placed to do using life course theory’s perspective and conceptual toolkit. Such concepts as trajectories or pathways, critical transitions, cohort effects, subcohorts and subcohort effects, cumulative disadvantage and intracohort differentiation are especially useful for identifying and examining factors shaping male aging across the life and into later years while remaining attentive to intervening factors that modify or amplify the impacts of social change on minoritized groups of gay men.

However, life course theory does not always neatly capture the complexities of gay male aging. While some applying the life course paradigm privilege early over later-life life course events, assuming that younger person’s developmental stage makes early-life historical contexts and events more formative and thus more consequential for later life, gay male aging – and LGBTQ aging more widely – demonstrates a wider range of critical moments across the life course when the intersection of biography and social change occurs. Moreover, life course theory’s traditional reliance on a heteronormative life course (e.g., one in which young adulthood offers social and financial benefits through heterosexual, legally recognized marriage) elides the cumulative disadvantage produced by the exclusion of gay men from these resources that the institutions supporting this life course provide. Thus, gay male aging studies can responsibly further both life course theory and social gerontology by expanding their understanding of ‘the life course’ and of the range of transitions (and these transitions’ timing, as above) that different cohorts experience as they move towards later life.

We have given examples of how the life course approach – specifically, its use of cohort and cohort effects - can uncover and document the impacts of these history/biography/structural context intersections. Life course theory can also, we suggest, point us to potential future gay male aging, as we can now see major historical events that are shaping gay men’s early and mid-life (anti-discrimination legislation passed in 2020, and anti-gay/anti-queer forces attacking the new queer generation). Charting the life-long impacts of these changes on older gay (and queer) men will doubtless be easier in future years, as increasing numbers of large-scale data sets are beginning to include questions on sexual orientation and gender identity – a luxury unavailable to scholars researching the current older gay male population.

## Author contributions

DR: Conceptualization, Writing – original draft, Writing – review & editing. JR-V: Conceptualization, Writing – original draft, Writing – review & editing.
