# Corrigendum: Beyond identity and generations: bringing life course theory to studies of older gay men

**DOI:** 10.3389/fsoc.2024.1445137

**Published:** 2024-07-24

**Authors:** Dana Rosenfeld, Jesus Ramirez-Valles

**Affiliations:** ^1^College of Liberal Arts and Sciences, University of Westminster, London, United Kingdom; ^2^School of Medicine, University of California San Francisco, San Francisco, CA, United States

**Keywords:** life course theory, gay male aging, queer aging, life course, male aging

In the published article, there was an error in the Funding statement. The funding source was erroneously omitted. The correct Funding statement appears below.

